# Language Barriers in the Delivery of Musculoskeletal Care and Future Directions

**DOI:** 10.1007/s12178-025-09986-3

**Published:** 2025-06-20

**Authors:** Edgar Garcia‒Lopez, Jamieson O’Marr, Rachel Gottlieb, Katherine Rebecca Miclau, Nirav Pandya

**Affiliations:** Orthopaedic Department, University of California San Francisco, 500 Parnassus Ave. Millberry Union MU 320 W, 94143 San Francisco, CA USA

**Keywords:** Non-English Language Preference, Language, Outcomes

## Abstract

**Purpose of Review:**

Examine how language barriers impact musculoskeletal care in the United States, particularly for patients with non-English language preference (NELP), and to highlight strategies to improve equity in orthopaedic outcomes.

**Recent Findings:**

In the U.S., 24.5 million individuals have NELP, yet few orthopaedic surgeons are proficient in non-English languages. NELP is consistently associated with worse postoperative outcomes, lower patient-reported outcome scores, and disparities in access to care. Studies have shown that language discordance contributes to miscommunication, reduced satisfaction, and suboptimal recovery. Despite federal mandates requiring interpreter services, access remains inconsistent, and many institutions fall short in providing language-concordant care. Recent research supports interventions such as professional interpreter use, language-concordant provider matching, multilingual discharge instructions, validated non-English PROMs, as well as the use of artificial intelligence.

**Summary:**

Language-related disparities in orthopaedic surgery are well-documented but under-addressed. Improving equity for patients with NELP will require system-wide efforts to standardize interpreter access, expand language-concordant care, and integrate emerging tools such as artificial intelligence to support communication across the surgical continuum.

## Introduction

In the United States, approximately 22% of residents speak a language other than English at home, a proportion that continues to rise [1]. From 1980 to 2019, the number of individuals speaking another language at home increased by 194%, from 23.1 million to 67.8 million, outpacing the overall population growth of 47%. In 2019, the five most common non-English languages spoken at home were Spanish (61.6%), Chinese (5.2%), Tagalog (2.6%), Vietnamese (2.3%), and Arabic (1.9%). Among these individuals, 37.6% have non-English language preference (NELP), defined as speaking English less than “very well”; however, this number varies greatly by language [2]. Certain states, including California, Nevada, New Mexico, Texas, Florida, New Jersey, and New York, have especially high linguistic diversity, with at least 30% of residents aged five years and older speaking languages other than English [1]. 

Despite the linguistic diversity among patients, few physicians, particularly in surgical specialties such as orthopaedics, report proficiency in non-English languages. According to a 2019 Association of American Medical Colleges survey, only 17.4% of surgeons reported proficiency in another language, with fewer surgeons routinely using this skill in patient care [3]. Data specific to orthopaedic surgeons in the U.S. is sparse; however, one study reported that only 5.1% of pediatric orthopaedic surgeons at academic centers are proficient in Spanish [4]. Another study of orthopaedic offices in California reported that, out of 35 surgeons, only 8.6% spoke sufficient Spanish to accommodate Spanish-speaking patients without the need for an interpreter [5]. 

NELP negatively impacts various aspects of healthcare, including fewer physician visits, a lack of consistent care, and an increased likelihood of foregoing necessary treatments [6]. In orthopaedics specifically, NELP has been linked to delayed surgeries, variations in anesthesia types, higher hospitalization costs, lower patient portal utilization, and decreased completion of patient-reported outcome measures [7]. Conversely, language concordance between patients and providers improves preventative and follow-up care adherence, reduces missed appointments and emergency department visits, decreases medication-related complications, and enhances patient satisfaction and perceived care quality [8–10]. 

Although NELP independently contributes to healthcare disparities, it frequently intersects with other social determinants of health, such as lower socioeconomic status, decreased insurance coverage, and lower educational attainment. Compared with English-language preferred (ELP) individuals, NELP individuals are significantly more likely to be uninsured or Medicaid dependent, have low income (19% vs. 12%), and have less than a high school education (39% vs. 7%) [11]. Additionally, NELP individuals are substantially more likely to have low health literacy (44.9%) than ELP individuals (13.8%) [12]. The effects of these factors, which can also be compounded by race, ethnicity, and citizenship status, can result in difficulties with healthcare navigation, adherence to treatment regimens, and comprehension of postoperative instructions.

Despite well-documented language-related barriers, the literature on effective interventions remains limited. This review aims to summarize the impact of language barriers on orthopaedic outcomes across subspecialties; explore the mechanisms through which language impacts postoperative outcomes; and explore practical solutions, such as improving language concordance, effective interpreter utilization, tailored preoperative education and discharge instructions, enhancing patient-reported outcome measure administration, and leveraging emerging technologies such as artificial intelligence.

## Impact of Language in Orthopaedics

Language impacts the full spectrum of healthcare for patients receiving orthopaedic care; from seeking care to postoperative outcomes, there are disparities associated with patient-provider language discordance that impact orthopaedic patients. Regarding access to care, NELP patients are less likely to be scheduled for an appointment when they call an orthopaedic office than their ELP counterparts [13]. They are less likely to utilize the online patient portal, which has been demonstrated to improve the quality of care and patient outcomes [14]. Patients presenting for the first time to an orthopaedic oncologist are more likely to present with metastatic disease if they are NELP [15]. Finally, delays from injury to surgery have been demonstrated in both pediatric ACL tears and distal radius fractures [16, 17]. 

During a surgical care episode, there are several differences in care between NELP and ELP patients. In hand surgery, patients transferred to a tertiary referral center for specialized care are more likely to need an interpreter than patients who are not transferred [18]. In total joint arthroplasty (TJA), patients who speak languages other than English or Spanish are less likely to receive neuraxial anesthesia [19]. NELP patients who undergo shoulder, hip, or knee arthroplasty have longer and more expensive hospitalizations and are more likely to be discharged to a skilled nursing facility [20–23]. In pediatric orthopaedic patients, NELP patients have a longer LOS and face delays in resolving barriers to discharge (caregiver education, durable medical equipment issues, transportation at discharge) and require more time to be medically cleared for discharge [24]. Following surgery, NELP patients are less likely to request refills on pain medications [25]. 

Regarding postoperative care, physical therapy is essential for optimizing recovery and improving functional outcomes. However, there is limited literature specifically examining the effects of language discordance in postoperative physical therapy within orthopaedics. Existing studies suggest that language barriers negatively impact the quality of physical therapy care [26, 27]. Both patients and physical therapists reported compromised treatment when professional interpreters were unavailable, leading to reduced communication, simplified instructions, and concerns about patient safety and effectiveness of care [26, 27]. These findings highlight the need for greater attention to language-concordant rehabilitation in orthopaedic postoperative care for NELP patients.

NELP patients also have higher postoperative complication rates. Following TJA, NELP patients have a greater risk of deep venous thrombosis, venous thromboembolism, and pneumonia [28]. Pediatric patients who underwent ACL reconstruction have a greater risk of graft tears requiring revision surgery [29]. NELP patients have higher postoperative pain scores and worse functional outcomes following TJA [30–33]. Importantly, NELP patients are less likely to have patient-reported outcome measures completed and documented in their charts [34]. Most of the literature concerning disparities in orthopaedic care comes from TJA; however, studies conducted in other subspecialties suggest that similar disparities exist across the field of orthopaedics.

## Why Does Language Affect Postoperative Outcomes??

Language barriers significantly impact postoperative outcomes, in large part by impairing communication between patients and providers. Misunderstandings about discharge instructions and postoperative care– such as rehabilitation protocols, activity restrictions, or symptom management– can lead to poorer adherence, which may result in delayed recovery and preventable complications [27, 35]. Language barriers intersect with broader social determinants of health, including socioeconomic status and insurance coverage, which can limit access to physical therapy and rehabilitation resources [36, 37]. In addition to language itself, cultural factors also play a critical role. Cultural perceptions of injury, pain tolerance, and the necessity of rehabilitation may differ widely, and failure to recognize or address these differences can further hinder recovery. Religious beliefs—an integral part of culture—may also shape healthcare decisions, such as pain management preferences or the willingness to undergo certain procedures [36, 38]. Considering these factors when providing care requires systemic institutional and policy-driven changes to ensure equitable postoperative care for linguistically diverse patients. However, among these challenges, communication barriers present an opportunity for more immediate intervention through improving language concordance, effective interpreter utilization, tailored preoperative education and discharge instructions, enhancing patient-reported outcome measure administration, and leveraging emerging technologies such as artificial intelligence.

## Potential Solutions and Tools

### Language-Concordant Care

Language-concordant care—when patients and providers speak the same language—has emerged as one of the most effective tools to bridge communication gaps and improve outcomes in populations with NELP [8–10]. In a pediatric surgery clinic, Dunlap et al. reported that Spanish-speaking families who received care from Spanish-proficient providers reported significantly better understanding and satisfaction than did those who relied on interpreters or received language-discordant care [39]. Similarly, studies across specialties have demonstrated that language-concordant care is associated with improved comprehension, increased trust, and better adherence to treatment plans [8– [10, 40, 41]. In orthopaedic surgery, where success hinges on clear patient understanding of surgical plans, postoperative expectations, and rehabilitation protocols, the consequences of language discordance can be significant.

Anecdotally, speaking to Spanish-speaking patients in Spanish fosters a deeper sense of trust and connection, making them more comfortable asking questions and sharing important details about their health. This trust is foundational in orthopaedic care, where shared decision-making and clear expectations are critical. However, despite the clear benefits of language concordance, there is a notable under-representation of Spanish speaking orthopaedic surgeons. The lack of diversity within orthopaedic surgery further contributes to this gap [42]. While not universally feasible, expanding access to language-concordant care— through multilingual provider recruitment through targeted recruitment of minority physicians via pipeline programs and improved retention through dedicated mentorship, formal documentation of provider language skills, and incorporation of language matching into clinical workflows—represents a scalable strategy to promote equity, improve surgical outcomes, and foster trust. Ultimately, patient‒physician language concordance may be the most effective solution for reducing disparities in orthopaedic surgery; however, the number of physicians who speak multiple languages is limited, thus other solutions must be explored.

### Interpreters

The use of professional interpreters is critical in reducing communication barriers for patients with NELP, particularly when language-concordant providers are unavailable. Section 1557 of the Affordable Care Act (ACA) mandates that healthcare providers “take reasonable steps to provide meaningful access to each individual with limited English proficiency” eligible for care or likely to be encountered in their programs. This includes not only patients, but also their families, spouses, or partners. Despite this federal requirement, interpreter services are often underutilized or inconsistently implemented in clinical practice, particularly in high-volume settings such as orthopaedic surgery.

Access to interpreters remains a major barrier in the delivery of equitable orthopaedic care for NELP. Greene et al. assessed access to orthopaedic appointments by language preference and reported no significant difference in appointment scheduling between simulated Spanish- and English-speaking patients (*p* = 0.8256) [5]. However, most Spanish-speaking callers—28 out of 35—were instructed to bring a friend or family member to interpret. Only four clinics offered a professional interpreter, and only three stated that the provider spoke enough Spanish to forgo one entirely. Asking patients to bring their own interpreter violates federal mandates under the ACA and places an undue burden on patients, many of whom may not have a family member available who is proficient in medical terminology or able to take time off work.

The type of interpreter used also significantly impacts the quality of patient–provider communication. A study from the University of Colorado compared family interpreters, telephone interpreters, and language-concordant providers [43]. Patients who relied on family members to interpret reported significantly lower satisfaction with their provider and the overall encounter than those with language-concordant care. Notably, there was no significant difference in satisfaction between telephone interpreters and language-concordant providers. While calling a professional interpreter may add time to the visit, these data reinforce that using certified interpreters, rather than family members, is essential to providing equitable, patient-centered care.

### Preoperative Education

Preoperative education is a crucial opportunity to communicate surgical expectations, risks, and benefits, as well as to reduce anxiety. This is particularly important for NELP patients, who may be in new and uncomfortable environments. From the initial consultation to informed consent and preoperative instructions, such as arrival times and NPO status, multiple points along the care continuum are vulnerable to failures in communication, shedding light on potential areas for improvement. The literature has demonstrated that NELP patients are less likely to have documented informed consent than ELP patients are [44] and are more likely to experience delays in care [45, 46]. 

To address these disparities, proposed strategies include ensuring interpreter access at all stages of care, including preoperative phone calls, consent discussions, and the procedural visit itself [47, 48]. Comprehensive programs that combine provider education with increased access to professional interpreters have shown success in certain surgical settings and offer a model for broader implementation [48]. A multifaceted approach that incorporates standardized multilingual education protocols, provider training, and the cautious use of AI-driven translation tools is essential for improving preoperative education and ensuring equitable surgical care for NELP patients.

### Discharge Instructions

Effective communication at the time of discharge—regarding follow-up care, medication regimens, return precautions, and more—is critical to ensuring high-quality, efficient patient care. This is especially true in surgical subspecialties such as orthopaedic surgery, where patients are frequently discharged with complex pain management plans, wound care instructions, mobility restrictions, and rehabilitation protocols that can significantly impact outcomes. For NELP patients, the discharge period can be particularly distressing, often marked by gaps in understanding that may lead to poorer outcomes, including readmissions and inadequate pain control [22, 49–52]. Emerging data suggest that providing language-concordant discharge instructions, whether written or verbal, can help mitigate these disparities and improve clinical outcomes [53, 54]. 

Despite this growing body of evidence and the requirement under the ACA to provide language-concordant materials, many hospitals continue to fall short in delivering these services [55, 56]. Ensuring that NELP patients receive high-quality, language-concordant discharge instructions is a vital step toward improving outcomes in this population. Strategies to achieve this include increasing access to professional translation services, integrating multilingual discharge templates into electronic medical records (EMRs), and enhancing provider awareness around culturally and linguistically appropriate care [57]. Language-concordant discharge instructions are an essential tool in promoting safe and equitable transitions of care.

### Patient Reported Outcome Measures (PROMs)

Patient-reported outcome measures (PROMs) are becoming increasingly prevalent in healthcare, and orthopaedics is no exception. Their use is being incorporated into clinical practice guidelines to inform treatment decisions and even, in some cases, to guide reimbursement [58]. PROMs help place the focus firmly on the patient and their lived experience, thereby promoting patient-centered care and supporting shared decision-making [59, 60]. When properly implemented, this emphasis on patient-centered care has the potential to improve the quality of care—particularly for patients who may have difficulty communicating with providers in more traditional ways. However, many current PROMs do not accurately capture outcome information for patients from diverse language backgrounds [61]. 

In the United States, based on the limited number of studies that report demographic data, PROMs have overwhelmingly been developed and validated among highly educated, English-speaking patients [62–65]. Many national and international organizations have emphasized the importance of ensuring proper cultural and linguistic adaptation of PROMs prior to implementation, given the significant risks associated with non-validated scores. As PROM use continues to grow, improperly adapted measures could inadvertently create new systemic barriers [66–68]. Even when PROMs are implemented, recent orthopaedic literature has shown that NELP patients and those of lower socioeconomic status complete PROMs at significantly lower rates [34]. These findings underscore the critical need for both linguistic and cultural adaptation, as well as increased system-level support to promote PROM completion among linguistically diverse populations. Despite these challenges, when PROMs are appropriately adapted and implemented, they can serve as a promising means for NELP patients to express their concerns and report outcomes efficiently and effectively to orthopaedic providers.

### Artificial Intelligence (AI)

Artificial intelligence (AI) is an area of rapid expansion in healthcare translation, offering promising solutions to overcome language barriers and enhance orthopaedic patient care. Recent governmental initiatives, such as California Governor Gavin Newsom’s Executive Order N-12-23 and the California Health and Human Services Agency’s (CalHHS) request for innovative ideas (RFI 2 #29672) on generative AI, highlight the growing recognition of AI’s potential to improve health equity through accurate, culturally sensitive, and cost-effective translations [69–71]. 

A systematic review of nine studies from 2019 to 2024 revealed that AI-powered translation platforms demonstrated high accuracy (83–97.8%) in translating from English to other languages but significantly lower accuracy (36–76%) in translating from other languages into English [72]. Patient satisfaction with AI translations was consistently high (84–96.6%), whereas clinicians reported lower satisfaction (53.8–86.7%). Clinician hesitancy stemmed primarily from concerns about translation quality, reliability, respectfulness, and potential clinical misunderstandings [72]. Nevertheless, ongoing technological advancements, increased integration into clinical workflows, and expanded research into AI translation platforms could mitigate these concerns and encourage broader adoption in orthopaedics.

Specific AI platforms have already been effectively integrated into clinical workflows. Implementing RadTranslate, an application that provides multilingual radiology examination audio instructions, resulted in reduced strain on medical interpreters and more reliable average examination lengths [73]. These platforms are particularly promising in orthopaedics, where clear and immediate instructions during diagnostic imaging and treatment planning can increase patient comprehension, adherence, and outcomes.

Additionally, AI documentation platforms, such as Ambience, utilize machine learning to deliver real-time scribe-like transcription to reduce clinicians’ documentation burdens. Ambience also has the capacity to facilitate multilingual transcription by automatically generating English clinical documentation from encounters conducted in commonly spoken U.S. languages (e.g., Spanish, Mandarin) [74]. The Permanente Medical Group (TPMG) implemented Ambient AI technology in October 2023, successfully engaging 3,442 of 10,000 eligible physicians across 303,266 patient encounters within ten weeks [74]. The results revealed a significant, dose-dependent reduction in after-hours EMR documentation time and notetaking during patient encounters. A quality assessment of 35 randomly selected transcripts achieved excellent outcomes, averaging 48 out of 50 on the modified 9-item Physician Documentation Quality Instrument (PDQI-9) instrument, although a few instances of AI-generated inaccuracies (“hallucinations”) were noted.

Despite these potential benefits, significant barriers persist for widespread AI implementation. Regulations currently limit the use of Ambience AI scribes to English-only clinical encounters due to certified medical interpretation requirements [74]. While intended to maintain translation quality standards, such restrictions may inadvertently increase documentation burdens for multilingual clinicians and discourage care for non-English-speaking patients in settings utilizing AI documentation. Rapid technological evaluation, advancement, and policy adjustments are urgently needed to enable equitable integration of AI transcription technologies in multilingual clinical environments.

Other inherent risks of using AI programs in documentation include significant translation errors, especially in less frequently used languages with limited training resources, and data security vulnerability, possibly leading to non-consensual disclosure of protected health information [75]. These risks highlight the need for robust oversight frameworks. Seattle Children’s Hospital has proposed an approach that includes the identification of the clinical context (time-sensitive versus nontimed-sensitive), the customization of AI models via locally translated resources validated by experts, active patient and family engagement, and continuous evaluation to ensure translation accuracy, safety, and patient relevance [75]. One such framework can be specifically applied to integrating AI technology in multilingual clinical encounters and patient materials to ensure equitable implementation.

Ultimately, implementing AI-based translation tools within orthopaedics shows promise for reducing language disparities and improving patient-provider communication. The successful development and implementation of AI-based translation tools will require interdisciplinary collaboration among clinicians, healthcare organizations, technology developers, linguists, and patients.

## Conclusion

While language-related barriers in orthopaedic care are well recognized, evidence-based interventions remain limited. This review highlights the significant impact of language barriers on postoperative outcomes across orthopaedics and outlines key mechanisms by which language influences patient care. We explore strategies to address language-related barriers, including improving language concordance, optimizing interpreter use, tailoring preoperative education and discharge instructions, adapting patient-reported outcome measures, and leveraging emerging technologies such as artificial intelligence. Together, these approaches underscore the crucial need for targeted, system-level interventions to advance equity and improve outcomes for NELP patients.

## Key References

- Torres RB et al. Is Limited English Proficiency Associated With Differences in Care Processes and Treatment Outcomes in Patients Undergoing Orthopaedic Surgery? A Systematic Review. Clin Orthop Relat Res. 2024;482:1374–90. This systematic review of 29 studies involving over 360,000 patients is the most comprehensive analysis to date of how NELP impacts musculoskeletal care. It demonstrates that NELP patients are more likely to experience delays in surgical treatment, reduced patient engagement, lower rates of patient-reported outcome collection, and non-home discharge—all key concerns in orthopaedic surgery.
- Ngo- Metzger Q et al. Providing high-quality care for limited English proficient patients: the importance of language concordance and interpreter use. J Gen Intern Med. 2007;22 Suppl 2:324–30. A widely cited, foundational study that established the importance of using trained medical interpreters and language-concordant providers to improve communication, reduce medical errors, and enhance overall care quality for NELP patients. It serves as a cornerstone for many language access policies in U.S. healthcare systems.
- Sobel AD et al. Evaluation of Spanish Language Proficiency and Resources Available in Academic Pediatric Orthopaedic Centers. J Pediatr Orthop. 2020;40:310–313. This cross-sectional survey of academic pediatric orthopaedic programs found that only 5.1% of faculty were proficient in Spanish, and just 34.8% of institutions provided online Spanish-language educational materials. The study highlights major structural barriers to providing linguistically appropriate orthopaedic care.
- Greene NE et al. Access to Orthopaedic Care for Spanish-Speaking Patients in California. JBJS. 2019;101:e95. Using simulated patient calls to 35 orthopaedic practices, this field study found that the vast majority of Spanish-speaking callers were told to bring their own interpreter, with only a small fraction offered professional interpretation. These findings point to critical lapses in legal compliance and infrastructure for language access in musculoskeletal clinics.
- Chonde DB et al. RadTranslate: An Artificial Intelligence-Powered Intervention for Urgent Imaging to Enhance Care Equity for Patients With Limited English Proficiency During the COVID-19 Pandemic. J Am Coll Radiol. 2021;18:1000–8. This innovative study describes the development of RadTranslate, an AI-based tool that delivers standardized, multilingual imaging instructions. The intervention improved communication with LEP patients, reduced reliance on live interpreters, and increased workflow efficiency—demonstrating the potential of AI to enhance care equity in high-volume clinical settings.

## Data Availability

No datasets were generated or analysed during the current study.
